# Role of Adipokines in the Association between Thyroid Hormone and Components of the Metabolic Syndrome

**DOI:** 10.3390/jcm8060764

**Published:** 2019-05-30

**Authors:** Alessandro P. Delitala, Angelo Scuteri, Edoardo Fiorillo, Edward G. Lakatta, David Schlessinger, Francesco Cucca

**Affiliations:** 1 Istituto di Ricerca Genetica e Biomedica (IRGB), Consiglio Nazionale delle Ricerche, c/o Cittadella Universitaria di Monserrato, 09042 Cagliari, Italy; edoardofiorillo@gmail.com (E.F.); fcucca@uniss.it (F.C.); 2 Department of Medicine Azienda Ospedaliero-Universitaria di Sassari, 07100 Sassari, Italy; 3 Department of Clinical and Experimental Medicine, University of Sassari, 07100 Sassari, Italy; d341elefante@virgilio.it; 4 Laboratory of Cardiovascular Science, National Institute on Aging, National Institute of Health, Baltimore, MD 21224, USA; LakattaE@grc.nia.nih.gov; 5 Department of Health and Human Services, National Institute on Aging, NIH, Baltimore, MD 21224, USA; SchlessingerD@grc.nia.nih.gov; 6 Department of Biomedical Sciences, University of Sassari, 07100 Sassari, Italy

**Keywords:** free thyroxine, thyrotropin, metabolic syndrome, leptin, adiponectin

## Abstract

Metabolic syndrome (MS) increases cardiovascular risk. The role of thyroid hormone on components of MS is unclear. We analyzed a sample of 4733 euthyroid subjects from SardiNIA study. In female thyrotropin (TSH) was significantly and positively associated with triglycerides (Standardized regression coefficients (*β*) = 0.081, *p* < 0.001). Free thyroxine (FT4) was positively associated with HDL (*β* = 0.056, *p* < 0.01), systolic blood pressure (SBP) (*β* = 0.059, *p* < 0.001), diastolic blood pressure (DBP) (*β* = 0.044, *p* < 0.01), and fasting glucose (*β* = 0.046, *p* < 0.01). Conversely, FT4 showed a negative association with waist circumference (*β* = −0.052, *p* < 0.001). In TSH was positively associated with triglycerides (*β* = 0.111, *p* < 0.001) and FT4 showed a positive association with DBP (*β* = 0.51, *p* < 0.01). The addition of leptin and adiponectin to the regression models did not substantially change the impact of thyroid hormones on components of MS. Our data suggest that, even within the euthyroid range, excess of truncal adipose tissue is associated with variations in FT4. Leptin and adiponectin exert an additive effect rather than a causal effect. Additional studies should be performed to determine the clinical significance of this finding.

## 1. Introduction

Metabolic syndrome (MS) is characterized by a cluster of cardiovascular risk factors, which increases the risk of cardiovascular disease (CVD) [1,2,3]. A clinical definition of the MS has been developed in the last two decades with the purpose of identifying those individuals at increased risk of these diseases in order to put in place preventive measures that can reduce this risk [4]. These risk factors include impaired glycemia, raised blood pressure, elevated triglyceride levels, low high-density lipoprotein cholesterol levels (HDL) and abdominal obesity (which is highly correlated with insulin resistance). The National Cholesterol Education Program (NCEP) Adult Treatment Panel III (ATP III) guidelines define MS as having three of these 5 modifiable risk factors. Once CVD or diabetes develops, the MS is often present, and the number of components of the MS contributes to disease progression and risk [5].

Thyroid hormone is an important regulator of cardiac function and changes in its concentration are associated with several alterations in cardiovascular hemodynamic. Metabolic changes found in MS are similar to those seen in overt hypothyroidism [6], but it has been still a concern for subclinical disorders [7,8]. Evidences suggest that disorders of the thyroid gland are associated with alterations in lipid metabolism, blood pressure, and increased cardiovascular risk [9,10]. Recent studies have shown that thyroid dysfunction can induce insulin resistance, but the evidences are not univocal [11]. Thus, MS shared common features with thyroid disorders, both being a risk factor of cardiovascular disease. MS has been associated with subclinical thyroid disease in adults due to the effect of thyroid function on cardiovascular dysfunction, lipid and glucose metabolism, and blood pressure [12]. There are few studies addressing the relation between thyroid function and MS in euthyroid subjects. Some authors reported an association between thyrotropin (TSH) and MS [13,14], some reported an association with free thyroxine (FT4) [15,16] and others did not find any [17,18]. The role of adipokines, leptin and adiponectin in particular, on metabolic syndrome is well acknowledged. Leptin has been found increased in subjects with hyperinsulinemia and type 2 diabetes, and showed a positive association with triglycerides, systolic and diastolic blood pressure [19]. On the other hand, adiponectin, whose levels are inversely related with visceral fat, has anti-atherogenic and anti-diabetic properties. Reduced levels are associated in some obesity-related disorders including hypertension [20] and type 2 diabetes mellitus [21]. Recently, leptin-adiponectin ratio (l/a) emerged as a good predictor of MS and type 2 diabetes, stronger than leptin and adiponectin alone [22].

In this study we aimed to clarify the relation between normal thyroid function, leptin and adiponectin with components of MS using a large cohort of euthyroid subjects from the general population.

## 2. Experimental Section

### 2.1. Participants and Data Recorded

The cohort is from the SardiNIA study, a population-based survey that investigates hundreds of genetic and phenotypic age-related traits [23]. Briefly, from 2001–2004, all residents in four towns of Lanusei valley (Lanusei, Arzana, Ilbono, and Elini) aged 14 years and older were invited to participate. In all, 6148 subjects were recruited, approximately 62% of the eligible population. For our analyses, subjects not being euthyroid and those using thyroid medications were excluded, as well as subjects taking glucose lowering medication, subjects taking lipid-lowering drugs and those who were on antihypertensive therapy, yielding a sample of 4733 subjects.

Each participant provided their informed consent. All study methods were conducted according to the principles expressed in the Declaration of Helsinki and were approved by the governing Ethics Committee, Azienda Sanitaria Locale (ASL 4).

### 2.2. Laboratory Measurements

Blood venous samples were drawn between 7 and 8 a.m. after an overnight fast. Serum samples were stored at −80 °C until use. TSH assessed with the Siemens TSH assay (Immulite 2000, Siemens, Healthcare Diagnostics Inc., Tarrytown, NY, USA) according to manufacturer’s instructions. The method is a solid-phase, two-site chemiluminescent immunometric assay (normal range 0.4–4.0 μIU/mL). FT4 was measured with the Siemens FT4 assay (Immulite 2000, Siemens, Healthcare Diagnostics Inc., Tarrytown, NY, USA). The method is a solid-phase, enzyme labelled chemiluminescent competitive immunoassay (normal range 0.89–1.76 ng/dL).

Plasma triglycerides and total cholesterol were determined by an enzymatic method (Abbott Laboratories ABA-200 ATC Biochromatic Analyzer, Irving, TX, USA). High-density lipoprotein (HDL) cholesterol was determined by dextran sulphate–magnesium precipitation [24]. Low-density lipoprotein (LDL) cholesterol was estimated by the Friedewald formula [25], calculated as follows: LDL cholesterol = total cholesterol − (HDL cholesterol + (triglycerides/5)). Fasting plasma glucose concentration was measured by the glucose oxidase method (Beckman Instruments Inc., Fullerton, CA, USA). Leptin and adiponectin (human serum adipokine—panel B; Lincoplex kit—Linco Research, Inc., 14 Research Park Dr., St. Charles, MO 63304, USA: Cat. # HADK2-61K-B) were measured with a multiplex testing Luminex Model no. Luminex 200 IS Serial No. LX10006265401 (Luminex Corporation, Austin, TX, USA).

### 2.3. Definitions

Euthyroidism was diagnosed in subjects with TSH within the reference range (0.4–4.0 μIU/mL), while not taking any thyroid therapy. Waist circumference (cm) was measured on bare skin between the 10^th^ rib and the iliac crest at the end of normal expiration. The homeostasis model assessment (HOMA) index was calculated as fasting glucose (mg/dL) times fasting insulin (mU/liter) divided by 405 [26].

The MS criteria was considered according to ATP III [27] and defined as the presence of at least three of the following five items: (1) abdominal obesity, defined as waist circumference in men greater than 102 cm and in women greater than 88 cm; (2) serum triglycerides ≥ 150 mg/dL; (3) serum HDL less than 40 mg/dL in men and less than 50 mg/dL in women; (4) systolic blood pressure (SBP) ≥ 130 mmHg or diastolic blood pressure (DBP) ≥ 85 mmHg; (5) fasting glucose ≥ 110 mg/dL.

Overweight was defined by the presence of BMI ≥ 25 Kg/m^2^.

### 2.4. Statistical Analysis

Normality was tested by the Shapiro-Wilk test for all the continuous variables under study. Because all of them had non-normal distribution nonparametric tests were used. Accordingly, median and interquartile range (IQR) were utilized as summary measures. Differences among male and female in continuous variable were tested using Kruskal-Wallis test. Fisher’s exact test was used to test differences in frequency. Linear regression models were performed to assess the association between components of metabolic syndrome (dependent variable) and thyroid hormone. Analyses were conducted for male and female, testing separately TSH and FT4. We created three different models: model 1, was adjusted for age, overweight, and HOMA-index; model 2 adjusted for age, overweight, HOMA index, leptin and adiponectin; model 3 adjusted for age, overweight, HOMA, leptin-adiponectin ratio. We also tested the interactive effect of thyroid hormone and cytokines on the components of metabolic syndrome entering appropriate interaction terms in all the analysis (TSH-by-leptin, TSH-by-adiponectin, TSH-by-l/a, FT4-by-leptin, FT4-by-adiponectin, FT4-by-l/a).

Results of regression models in the text were reported as standardized regression coefficients (*β*) and *p* value, which was set at <0.05 in Stata 12.0 (StataCorp LLC, College Station, TX, USA).

## 3. Results

**Figure 1:**
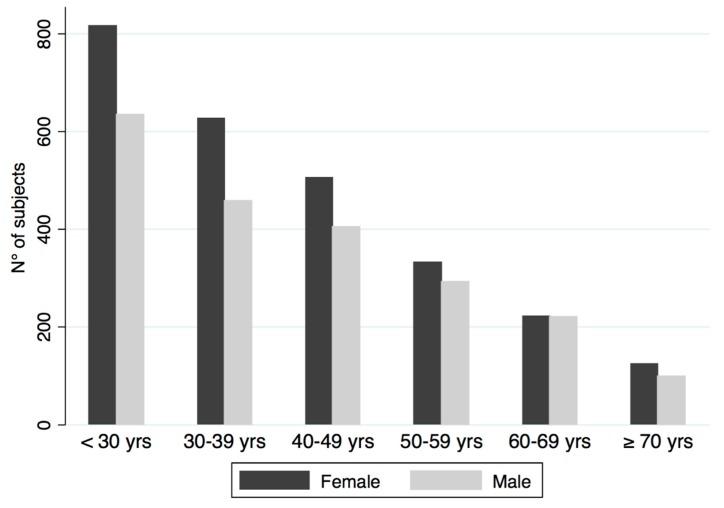
Age decades accordingly to gender.

Descriptive statistics, stratified by gender, are provided in Table 1 and Figure 1. The overall prevalence of metabolic syndrome in the sample was 4.3%. Both male and female participants had comparable age, total cholesterol, and fasting insulin levels. Females had lower BMI and waist circumference and had lower blood pressure values (*p* < 0.001 for all). On the contrary males had a worst lipid status: higher LDL, triglycerides, and lower HDL (*p* < 0.001 for all). Females had higher TSH values, showed lower fasting glucose concentration (*p* < 0.001 for all) and a lower HOMA index (*p* < 0.01). Finally, Female had higher leptin and adiponectin levels as compared to male (*p* < 0.001 for both).

Table 2 showed the comparison between thyroid hormones and cytokines accordingly to the presence of MS, stratified by gender. Subjects with MS had increased leptin and l/a and reduced adiponectin levels.

Table 3 showed the result of multiple linear regression models in female. In model 1, adjusted for age, sex, overweight, and HOMA index, TSH was significantly and positively associated with triglycerides (*β* = 0.081, *p* < 0.001). On the contrary, waist circumference, SBP, DBP, and fasting glucose were not associated with TSH. Addition of the cytokines and their ratio to the regression models did not substantially change the impact of TSH levels on triglycerides (*β* = 0.081, *p* < 0.001). As for FT4, model 1 showed that it was positively associated with HDL (*β* = 0.056, *p* < 0.01), SBP (*β* = 0.059, *p* < 0.001), DBP (*β* = 0.044, *p* < 0.01), and fasting glucose (*β* = 0.046, *p* < 0.01). Conversely, FT4 showed a negative association with waist circumference (*β* = −0.052, *p* < 0.001). Addition of the leptin and adiponectin to the multivariable regression model 1, attenuated the impact of FT4 on HDL (*β* = 0.048, *p* < 0.05), waist circumference (*β* = −0.044, *p* < 0.01) and total cholesterol (*β* = 0.031, *p* = ns), while enhanced its effect on DBP (*β* = 0.044, *p* < 0.01), fasting glucose (*β* = 0.050, *p* < 0.01). In Model 3, l/a was negatively associated with HDL (*β* = −0.135, *p* < 0.001) and showed a positive association with waist circumference (*β* = 0.150, *p* < 0.001), SBP (*β* = 0.047, *p* <0.01) and DBP (*β* = 0.079, *p* < 0.001).

Results of multiple linear regression models in male are showed in Table 4. In model 1, TSH was positively associated with triglycerides (*β* = 0.111, *p* < 0.001). After addition of cytokines to the model, we observed a reduced impact of TSH on the dependent variables (triglycerides: *β* = 0.106, *p* = < 0.001). FT4 showed a positive association with DBP (*β* = 0.51, *p* < 0.01), which was not modified after the addition of leptin and adiponectin to the model. In model 3, FT4 showed the same trend of previous models and l/a had a positive association with waist circumference (*β* = 0.142, *p* < 0.001) and triglycerides (*β* = 0.047, *p* < 0.05) and a negative correlation with HDL (*β* = −0.141, *p* < 0.001)

We further tested whether thyroid hormone increased the risk of MS. As reported in Table 5, TSH nor FT4 predicted the risk of MS.

Finally, we tested interaction terms in all statistical models. Interaction between thyroid hormones (FT4 or TSH) with cytokines (leptin, or adiponectin, or l/a) was not significant in male as well in female.

## 4. Discussion

In this study we aimed to clarify the relation between TSH and FT4 levels with the component of the MS in euthyroid male and female without thyroid diseases. The present population-based survey indicates a clear gender effect on the association between components of MS and thyroid hormones. Indeed, in female five out six components were associated with FT4 and 1 out 6 with TSH. On the contrary, in male, only DBP was associated with FT4, while TSH was associated with LDL and triglycerides.

Hormones play a pivotal role in adipocytes metabolism [28,29], and thyroid hormones upregulate many metabolic pathways relevant for resting energy expenditure. In particular, hyperthyroidism is associated with weight loss despite increased appetite and elevated metabolic rate, whereas hypothyroidism is related to a modest weight gain and decreased metabolic rate. Less clear are the potential mechanisms underlying the association between thyroid function and body fat in euthyroid individuals, although TSH and BMI might be positively correlated. Several cross-sectional studies have reported associations of body fat with higher serum TSH and T3 and lower T4 among euthyroid individuals [30,31], although with some inconsistencies probably related to different methodologic approaches and/or small samples of participants [32]. Several causes of increased TSH levels in obesity have been suggested. Apart from the coexistence of autoimmune thyroiditis, which are highly prevalent in the general population [33], a neuroendocrine dysfunction mediated by the adipocyte derived leptin has been postulated as a cause of elevated TSH levels in obesity [34,35,36]. A decreased negative feed-back due to partially bio-inactive TSH or the presence of a partial hormone resistance due a decrease in T3 receptors have both been suggested, although experimental studies providing this hypothesis are missing and therefore these concepts are very speculative.

Many studies have assessed the association between thyroid dysfunction and the MS. Some studies concentrated only on serum TSH levels [13,37] or evaluated MS components individually and not the syndrome as whole [14]. However, variable correlations between serum TSH, thyroid hormones and components of the MS have been found in these cross-sectional studies [17,38]. Other studies suggested an increased risk of MS in patients with elevated TSH levels [39,40], whereas FT4 levels have been shown to be associated with four of the five components of the MS [15]. More recently, no significant associations between serum TSH levels and prevalence of MS or individual components of the MS in obese and overweight individual have been reported [41]. However, no serum FT4 levels were available in this study and no associations between FT4 and individual component of the MT could be done.

We found a positive association between FT4 and blood pressure in both female and male, although the latter only with DBP. An association between FT4 and blood pressure has been reported in several studies and could be due to the effect of thyroid hormone on cardiovascular system. Indeed, excess of thyroid hormone increases caused a decrease of systemic vascular resistance and an increase of heart rate and contractility, thus increasing SBP [9]. In addition, a possible endothelial dysfunction has been postulated by some authors [42]. However, the duration of this effect is still not clear, as suggested by Itterman who found an association between subclinical hyperthyroidism and current, but not incident, hypertension in a pooled data analysis [43]. A synergistic effect of increased arterial stiffness has been also postulated [44,45,46].

In our study we found a positive association between TSH and triglycerides, both in male and female. The action of thyroid hormone on lipid metabolism has been well documented and is due to different mechanisms. Thyroid hormone stimulates the transcription of the low-density lipoprotein cholesterol (LDL) receptor gene, induces the hepatic expression of hydroxymethylglutaryl coenzyme A reductase (HMG-CoA), increases the expression of the sterol regulatory element-binding protein-2 (SREBP-2) that in turn modulates expression of LDL receptor. Thyroid hormone may contribute to maintain cholesterol homeostasis through the conversion of cholesterol to bile acids and subsequent faecal, and also decreases cholesterol ester transfer protein (CEPT) concentrations, acting in HDL metabolism. Finally, lipoprotein lipase activity is increased by thyroid hormone [10]. The gender effect on this association has been recently studied [47,48] and our findings are in line with previous reports.

We also showed that FT4 has a direct and positive association with fasting glucose in female. While the role of thyroid hormone on glucose metabolism has been well documented, the reason of this gender effect will require more studies, although it is logical to believe a central role of sex hormones [49], as clinically evident in women with polycystic ovary syndrome who has at increased risk to develop insulin resistance and diabetes [9].

Finally, we also found that serum FT4 concentration in euthyroid female was negatively associated with waist circumference in female, suggesting that lower FT4 might be related to visceral obesity, thus predisposing to insulin resistance. Thyroid hormone modulates the activity of different metabolic pathways associated with the basal metabolic rate, such as uncoupling of cellular metabolism from adenosine triphosphate (ATP) synthesis or modifying metabolic process downstream from mitochondria [50].

Previous studies hypothesized that the effect of thyroid hormone on MS could be associated with abnormal adipocytokine production. Our study clearly showed that the effect of the most abundant cytokines (leptin and adiponectin) have an addictive and independent effect on this association, rather than a causal effect. We can only speculate that other mechanisms (e.g., different cytokines) may explain the role of thyroid hormones on MS. Similarly to previous studies, we also demonstrated the importance of l/a on components of metabolic syndrome [51]. Indeed, we found that l/a are associated with almost all the components of MS although with gender difference.

The overall frequency of MS in our sample was lower than what already reported, but this is due to the tight exclusion criteria we used. Indeed, we did not include subjects taking glucose lowering and hypotensive medication as well as those on lipid-lowering drugs, thus excluding a high number of subjects that fulfil the ATP III criteria of MS. The different results observed in other studies may be caused by variations in the definition of MS, differences in sample size as well as thyroid status, since some studies reported a higher prevalence of subclinical hypothyroidism, whereas our population was clearly euthyroid and without subclinical thyroid diseases.

There are some limitations in this study. Due to the cross-sectional design, we were limited in our ability to assess the timing of the association between the accumulation of body fat and variations in FT4 levels. Moreover, although waist circumference cannot accurately distinguish visceral fat from subcutaneous adipose tissue, thyroid hormones should be evaluated in relation to visceral adiposity to understand the relationships between thyroid hormones and insulin resistance. Further, all the subjects had only one assessment of TSH and FT4 (but not free triiodothyronine), thus potentially including individuals with non-thyroidal illness or transient abnormality. However, the strengths of our study include the relatively large sample size from a homogeneous population, and tight exclusion criteria.

In summary, we observe a clear gender effect on the association between thyroid hormones and the components of MS. Although changes in body fat are well known consequences of overt thyroid dysfunction, our data suggest that, even within the euthyroid range, excess of truncal adipose tissue is associated with variations in both TSH and FT4 levels. Leptin and adiponectin exert an additive effect in this association and not a causal effect. Experimental and/or further prospective studies should be performed in order to determine the clinical significance of our findings in euthyroid states.

## Figures and Tables

**Table 1:** Descriptive characteristics of cardiovascular risk factors of the sample.

	Female	Male	Total
*n*	2628	2105	4733
Age (yrs.)	37.6 (27.2–50.4)	39.1 (27.6–52.2)	38.3 (27.4–51.3)
BMI (Kg/m^2^)	23.1 (20.6–26.2)	25.5 (22.9–28.1)	24.2 (21.5–27.4) ^¶^
Waist (cm)	76.0 (70.0–85.0)	89.0 (81.0–96.0)	82.0 (73.0–92.0) ^¶^
SBP (mmHg)	117 (108–127)	127 (118–137)	120 (111–132) ^¶^
DBP (mmHg)	72 (69–80)	79 (70–86)	75 (70–82) ^¶^
Total cholesterol (mg/dL)	204 (177–231)	206 (174–238)	205 (176–234)
LDL (mg/dL)	120 (99–144)	127 (102–153)	123 (100–148) ^¶^
HDL (mg/dL)	67 (58–77)	57 (49–66)	63 (53–73) ^¶^
Triglycerides (mg/dL)	61 (45–88)	79 (54–120)	68 (49–101) ^¶^
Fasting glucose (mg/dL)	82.4 (76.6–89.0)	87.9 (81.6–95.7)	84.8 (78.1–92.2) ^¶^
Fasting insulin (μU/L)	6.4 (4.3–9.7)	6.5 (4.1–9.6)	6.4 (4.2–9.6)
HOMA-Index	1.3 (0.9–2.0)	1.4 (0.9–2.2)	1.3 (0.9–2.1) ^#^
TSH (μUI/mL)	1.7 (1.2–2.4)	1.5 (1.0–2.0)	1.6 (1.1–2.2) ^¶^
FT4 (ng/dL)	1.3 (1.2–1.4)	1.3 (1.2–1.4)	1.3 (1.2–1.4) ^¶^
Leptin (pg/mL)	7492 (3530–13052)	3080 (1250–5660)	4910 (2040–9970)
Adiponectin (mg/dL)	2.8 (1.9–3.8)	1.8 (1.2–2.6)	2.3 (1.5–3.4) ^¶^
Leptin/adiponectin	2640 (1046–5424)	1577 (583–3697)	2116 (778–4690) ^¶^
Metabolic syndrome (*n*, %)	94, 3.0%	108, 5.1%	202, 4.3%

Continuous data are given as median and interquartile range; categorical data are expressed as absolute and relative frequencies. Continuous variables were compared using Mann–Whitney U test. Categorical variables were compared with Pearson χ2 test. Abbreviations: BMI, body mass index; SBP, systolic blood pressure; DBP, diastolic blood pressure; LDL, low density cholesterol; HDL, high density cholesterol; HOMA, homeostatic model assessment; TSH, thyrotropin; FT4, free thyroxine. ^#^ *p* < 0.01; ^¶^ *p* < 0.001.

**Table 2:** Comparison between thyroid hormones and cytokines in subjects with and without metabolic syndrome, stratified by gender.

	Female		Male	
	MS	No MS	MS	No MS
Leptin (pg/mL)	1,3400 (6530–2,5080) ^¶^	7355 (3483–1,2798)	5840 (2310–1,0300) ^¶^	2980 (1194–5474)
Adiponectin (mg/dL)	2.29 (1.67–3.28) ^#^	2.79 (1.96–3.85)	1.52 (1.17–2.41) *	1.81 (1.26–2.66)
l/a	5742 (1355–1,3463) ^¶^	2607 (1034–5276)	3703 (1355–6732)	1497 (569–3494) ^¶^
TSH (μUI/mL)	1.44 (0.84–2.26) ^#^	1.74 (1.19–2.40)	1.39 (1.05–2.00)	1.49 (1.03–2.05)
FT4 (ng/dL)	1.26 (1.16–1.40)	1.28 (1.18–1.40)	1.25 (1.13–1.37)	1.30 (1.19–1.43) ^#^

Data are presented as median and interquartile range. Variables were compared, separately for male and female, accordingly to presence/absence of MS using Mann–Whitney U test. Abbreviations: MS, metabolic syndrome; l/a, leptin/adiponectin ratio; TSH, thyrotropin; FT4, free thyroxine. * *p* < 0.05; ^#^ *p* < 0.01; ^¶^ *p* < 0.001.

**Table 3:** Association of thyroid function with components of the metabolic syndrome in female.

	Model	TSH	Leptin	Adiponectin	l/a	FT4	Leptin	Adiponectin	l/a
HDL	1	0.011	-	-	-	0.056 ^#^	-	-	-
	2	0.020	−0.059 ^¶^	0.156 ^¶^	-	0.048 *	−0.056 ^#^	0.153 ^¶^	-
	3	0.020	-	-	−0.138 ^¶^	0.052 ^#^	-	-	−0.135 ^¶^
Triglycerides	1	0.081 ^¶^	-	-	-	−0.021	-	-	-
	2	0.081 ^¶^	−0.034	−0.041 *	-	−0.021	−0.029	−0.041 *	-
	3	0.079 ^¶^	-	-	0.011	−0.021	-	-	0.014
Waist	1	0.002	-	-	-	−0.052 ^¶^	-	-	-
	2	−0.011	0.175 ^¶^	−0.042 ^¶^	-	−0.044 ^#^	0.173 ^¶^	−0.040 ^#^	-
	3	−0.006	-	-	0.152 ^¶^	−0.046 ^¶^	-	-	0.150 ^¶^
SBP	1	0.009	-	-	-	0.059 ^¶^	-	-	-
	2	0.007	0.062^¶^	0.025	-	0.059 ^¶^	0.065 ^#^	0.022	-
	3	0.009	-	-	0.044 ^#^	0.060 ^¶^	-	-	0.047 ^#^
DBP	1	0.024	-	-	-	0.044 ^#^	-	-	-
	2	0.023	0.083 ^¶^	0.013	-	0.046 ^#^	0.086 ^¶^	−0.011	-
	3	0.024	-	-	0.076 ^¶^	0.047 ^#^	-	-	0.079 ^¶^
Fasting glucose	1	−0.015	-	-	-	0.046 ^#^	-	-	-
	2	−0.016	−0.012	−0.055 ^#^	-	0.050 ^#^	−0.011	−0.058 ^#^	-
	3	−0.016	-	-	0.022	0.049 ^#^	-	-	0.024

Values are presented as β, standardized regression coefficients. Model 1, adjusted for age, overweight, and HOMA-index. Model 2, adjusted for age, overweight, HOMA, leptin, adiponectin. Model 3, adjusted for age, overweight, HOMA, leptin-adiponectin ratio. Abbreviations: TSH, thyrotropin; FT4, free thyroxine; HDL, high density cholesterol; SBP, systolic blood pressure; DBP, diastolic blood pressure; l/a, leptin-adiponectin ratio. * *p* < 0.05; ^#^ *p* < 0.01; ^¶^ *p* < 0.001.

**Table 4:** Association of thyroid function with components of the metabolic syndrome in male.

	Model	TSH	Leptin	Adiponectin	l/a	FT4	Leptin	Adiponectin	l/a
HDL	1	−0.042	-	-	-	−0.006	-	-	-
	2	−0.033	**−0.069** ^#^	**0.155** ^¶^	-	−0.022	**−0.070** ^#^	**0.157** ^¶^	-
	3	−0.029	-	-	**−0.139** ^¶^	−0.015	-	-	**−0.141** ^¶^
Triglycerides	1	**0.111** ^¶^	-	-	-	0.015	-	-	-
	2	**0.106** ^¶^	0.026	**−0.078** ^¶^	-	0.022	0.029	**−0.080** ^¶^	-
	3	**0.106** ^¶^	-	-	0.041	0.018	-	-	**0.047** *
Waist	1	0.008	-	-	-	−0.022	-	-	-
	2	0.001	**0.138** ^¶^	**−0.051** ^¶^	-	0.004	**0.139** ^¶^	**−0.051** ^¶^	-
	3	−0.003	-	-	**0.142** ^¶^	0.002	-	-	**0.142** ^¶^
SBP	1	0.007	-	-	-	0.033	-	-	-
	2	0.006	0.001	0.015	-	0.034	0.003	0.013	-
	3	0.007	-	-	−0.005	0.035	-	-	−0.004
DBP	1	0.010	-	-	-	**0.051** ^#^	-	-	-
	2	0.013	0.018	−0.015	-	**0.053** ^#^	0.019	−0.018	-
	3	0.013	-	-	0.022	**0.053** ^¶^	-	-	0.025
Fasting glucose	1	−0.028	-	-	-	0.016	-	-	-
	2	−0.024	−0.021	−0.022	-	0.020	−0.022	−0.023	-
	3	−0.024	-	-	−0.005	0.018	-	-	−0.005

Values are presented as β, standardized regression coefficients. Model 1, adjusted for age, overweight, and HOMA-index. Model 2, adjusted for age, overweight, HOMA, leptin, adiponectin. Model 3, adjusted for age, overweight, HOMA, leptin-adiponectin ratio. Abbreviations: TSH, thyrotropin; FT4, free thyroxine; HDL, high density cholesterol; SBP, systolic blood pressure; DBP, diastolic blood pressure; l/a, leptin-adiponectin ratio. * *p* < 0.05; ^#^ *p* < 0.01; ^¶^ *p* < 0.001.

**Table 5:** Effect of thyroid hormones and cytokine of metabolic syndrome: results of logistic regression.

	Model	TSH	Leptin	Adiponectin	l/a	FT4	Leptin	Adiponectin	l/a
Female	1	0.94 (0.71–1.24)	-	-	-	1.27 (0.37–4.38)	-	-	-
	2	0.89 (0.67–1.18)	1.00 (1.00–1.00)	0.78 (0.67–0.91) ^¶^	-	1.42 (0.40–5.05)	1.00 (1.00–1.00)	0.78 (0.67–0.91) ^¶^	-
	3	0.89 (0.67–1.18)	-	-	1.00 (1.00–1.00)	1.51 (0.43–5.31)	-	-	1.00 (1.00–1.00)
Male	1	1.26 (0.93–1.69)	-	-	-	1.83 (0.54–6.17)	-	-	-
	2	1.19 (0.87–1.63)	1.00 (1.00–1.00)	0.82 (0.67–1.00) *	-	2.19 (0.63–7.60)	1.00 (1.00–1.00)	0.80 (0.65–0.98) *	-
	3	1.20 (0.88–1.64)	-	-	1.00 (1.00–1.00)	1.79 (0.52–6.11)	-	-	1.00 (1.00–1.00)

Values are presented as Odds Ratio and 95% confidence interval. Model 1, adjusted for age, overweight, and HOMA-index. Model 2, adjusted for age, overweight, HOMA, leptin, adiponectin. Model 3, adjusted for age, overweight, HOMA, leptin-adiponectin ratio. Abbreviations: TSH, thyrotropin; FT4, free thyroxine; HDL, high density cholesterol; SBP, systolic blood pressure; DBP, diastolic blood pressure; l/a, leptin-adiponectin ratio. * *p* < 0.05; ^¶^ *p* < 0.001.
